# Soil phototroph community resilience comes from down under

**DOI:** 10.3389/fmicb.2025.1689042

**Published:** 2025-12-10

**Authors:** Brian Scott, Ferran Garcia-Pichel

**Affiliations:** 1 Center for Fundamental and Applied Microbiomics, Biodesign Institute, Arizona State University, Tempe, AZ, United States; 2 School of Life Sciences, Arizona State University, Tempe, AZ, United States

**Keywords:** microbial seed banks, soil microbiome resilience, phototroph, biocrust, undercrust

## Abstract

**Introduction:**

Seed banks are widely recognized as means of recovery following disturbance across diverse ecosystems, including soil. Phototrophic microbes, while common in surface soils, have not been considered within this context.

**Methods:**

We subjected a variety of topsoil photosynthetically-driven communities (biocrusts) to severe disturbance by scalping off the surface layer, thereby exposing the undercrust. We collected samples from the biocrust layer, the undercrust layer, and again the recovering biocrust layer after a 4-month period. Samples were analyzed for biopigments (chlorophyll a and scytonemin) and 16S rRNA gene copies and its sequence diversity.

**Results:**

Ribosomal gene counts and pigment analyses revealed consistently rapid recovery, as much as 52% content in 4 months. Recovery could be traced to dormant cyanobacterial “seeds” in the undercrusts. Alternative pathways for recovery, including natural or interventional inoculation and lateral spread, did not constitute a comparable force.

**Discussion:**

Our findings bring soil phototrophs within the ecological framework linking seed banks to resilience following disturbance. The overlooked role of undercrusts in prior research invites a reinterpretation of past studies and may inform new restoration strategies.

## 1 Introduction

Microbes, ubiquitous in soil, form universally complex communities that functionally underpin biogeochemical cycling and soil health (Madsen, 2011). To recover from local environmental disturbances, which affect their function, soil microbes benefit from prolific dispersal (Philippot et al., 2021), mediated by wind, overland water runoff, and animal vectors. Studies of “virgin” soil, new volcanic terrain or retreating glaciers, show that dispersal-driven bacterial re-colonization can be detected within months (Hadland et al., 2024), although this mechanism has inherent lag times, as evidenced by biogeographical distance-decay patterns (Barbour et al., 2023) and by the fact that full functional recovery is progressive and can last decades (Strauss et al., 2012). Proximal microbial seed banks, more effective if present, can also drive microbial community recovery (Lennon et al., 2021). The complex structure of soil supports seed banks through spatial refugia that enable long-lived microbial dormancy (Baho et al., 2012; Locey et al., 2016; Lennon and Jones, 2011). Such dormant, viable microbes can be found even into the deep biosphere (Shoemaker and Lennon, 2018).

Microbial dormancy describes a temporary state of extremely reduced metabolic activity (Greening et al., 2019) with arrested growth (McDonald et al., 2024). Metabolic activity leading to growth halts during dormancy, as demonstrated for photosynthesis (Harel et al., 2004) and the pentose phosphate pathway (Doello et al., 2025; Sawers, 2016) among cyanobacteria. Even in the absence of growth, a source of energy is needed to maintain basic cellular homeostasis and to repair unavoidable damage to proteins and nucleic acids, the “minimal maintenance energy” (Hoehler and Jorgensen, 2013; Lever et al., 2015). Importantly, dormant microbes remain viable and able to resuscitate, given the proper conditions, the fraction of cells remaining viable within a dormant population declining with time. The ability to resuscitate from dormancy requires preemptive accumulation of internal sources of energy (as reserve polymers) (Garcia-Pichel and Sala, 2022) and preemptive expression of genes that will be needed upon transition to growth (Rajeev et al., 2013).

Microbial seed banking and dispersal contribute significantly to the resilience of soils against losses of both diversity and function (Philippot et al., 2013). Where plant growth is restricted, such as in undisturbed arid or polar soils, phototrophic cyanobacteria are widespread in surface soils, becoming prominent and ecologically important. In these regions, terrestrial phototrophs take on many ecosystem-level functions such as primary production and erosion protection (Garcia-Pichel and Belnap, 2021). In drylands, terrestrial phototrophs form “biocrust” communities act as keystone members. For growth and basal maintenance, phototrophic microbes rely on radiant energy, which only penetrates the soil for about a millimeter (Garcia-Pichel, 2023), suggesting that they cannot take advantage of the refugia deep in the soil that heterotrophs might use to survive.

Because of their large aerial coverage, biocrust communities significantly contribute to global budgets of C and N (Elbert et al., 2012). These surface communities suffer widespread and diverse forms of anthropogenic disturbance, often catastrophic, from land use change, agriculture, ranching, urban sprawl, outdoor recreation, and, most recently, solar farm developments (Zaady et al., 2013; Belnap, 1993). Arid lands are already home to nearly 40% of the world’s population (Reynolds et al., 2007), and growing (Liu et al., 2019; Gilman and Wu, 2024), supporting more than 40% of global agricultural productivity (Wang et al., 2022; Elouafi, 2025). These numbers have been increasing (Ren et al., 2022). Global change models project that drylands will continue to gain dominance as a global force (Grunzweig et al., 2022), giving rise to a burgeoning field of biocrust restoration, usually by interventional inoculation with cultures or transplanted phototrophs (Heredia-Velásquez et al., 2023; Belnap, 1993; Antoninka et al., 2020; Chiquoine et al., 2016; Velasco Ayuso et al., 2017; Roncero-Ramos et al., 2022). Historically, biocrusts have been considered fragile and slow to recover from major disturbance, requiring decades in some cases (Belnap and Eldridge, 2003; Guida et al., 2023; Rubio and Lazaro, 2023). Still, conflicting reports of their recovery rates (Phillips et al., 2025) suggest an inadequate understanding of the systems’ complexities (Turner, 2021).

Below the biocrust proper and the photic zone is a heterotrophic zone of loose soil with an abrupt physical ecotone upper boundary. This “undercrust” has markedly different microenvironments, chemical composition, and microbial communities (Xu et al., 2023) from those in the biocrust. While influenced by leaching of micronutrients (Beraldi-Campesi and Garcia-Pichel, 2011) and organic carbon from the photosynthetically active layer above it (Young et al., 2022; Kut and Garcia-Pichel, 2024), the undercrusts share general characteristics with bulk desert soil: low microbial biomass and nutrient levels (Miralles et al., 2020; Garcia-Pichel et al., 2003). The undercrust is understood as little more than a physical substrate that benefits from exogenous inputs originating in the surface layer. Consequently, most investigations, including those focused on post-disturbance recovery, do not anticipate a role of the undercrust.

We set up a field experiment where we exposed the undercrust and monitored recovery. We completely removed the surface layer to mimic severe physical disturbances that may come from natural sources, such as ungulate trampling, erosional abrasion by overland flow, or anthropogenic sources such as recreational vehicle trampling. The experiment was conducted simultaneously in various locations in the lower Sonoran Desert that harbored different biocrust types. Our efforts were prompted by the preliminary observation of the swift recovery of natural biocrusts after they had been scalped to gather inoculum for the restoration of degraded farm soils. Transplanted to fallow farms, these inocula failed to grow. We hypothesized that natural soils must have some intrinsic properties (absent in farm soils) that afford them high resilience. Our results suggest that allochthonous dispersal or interventional inoculation contributes minimally to recovery and, instead, indicate that undercrust communities serve as the primary driver of recovery, originating from small but diverse and effective “seed” populations.

## 2 Methods

### 2.1 Field site locations

Test sites were spread out across central Arizona, United States, in the Sonoran Desert. The location was a former farm (32°54′7.94″N, 111°35′33.26″W), abandoned for at least 40 years, and now recovered to “natural” desert with mature biocrusts and typical vegetation (Scott et al., 2025). We establish four “core” experimental sites in this general area. Two had light cyanobacterial biocrusts (DG and LV), and two had dark cyanobacterial biocrusts (DD and ND). A fifth site, approximately 4 km south, supported a mixture of dark cyanobacterial and cyanolichen biocrusts (LC). Sites 25 and 31, approximately 50 km to the southeast, were dominated by lichens, with some bryophytes observed at 25. We also prepared test plots at a solar farm in Mesa, Arizona (site SF), where we had previously investigated soil recovery rates (Heredia-Velásquez et al., 2023). Recently fallowed farms, effectively devoid of biocrusts, served as experimental controls. Approximate site and farm locations are shown in Supplementary Figure S1.

### 2.2 Plot preparation and sample collection

**Figure 1:**
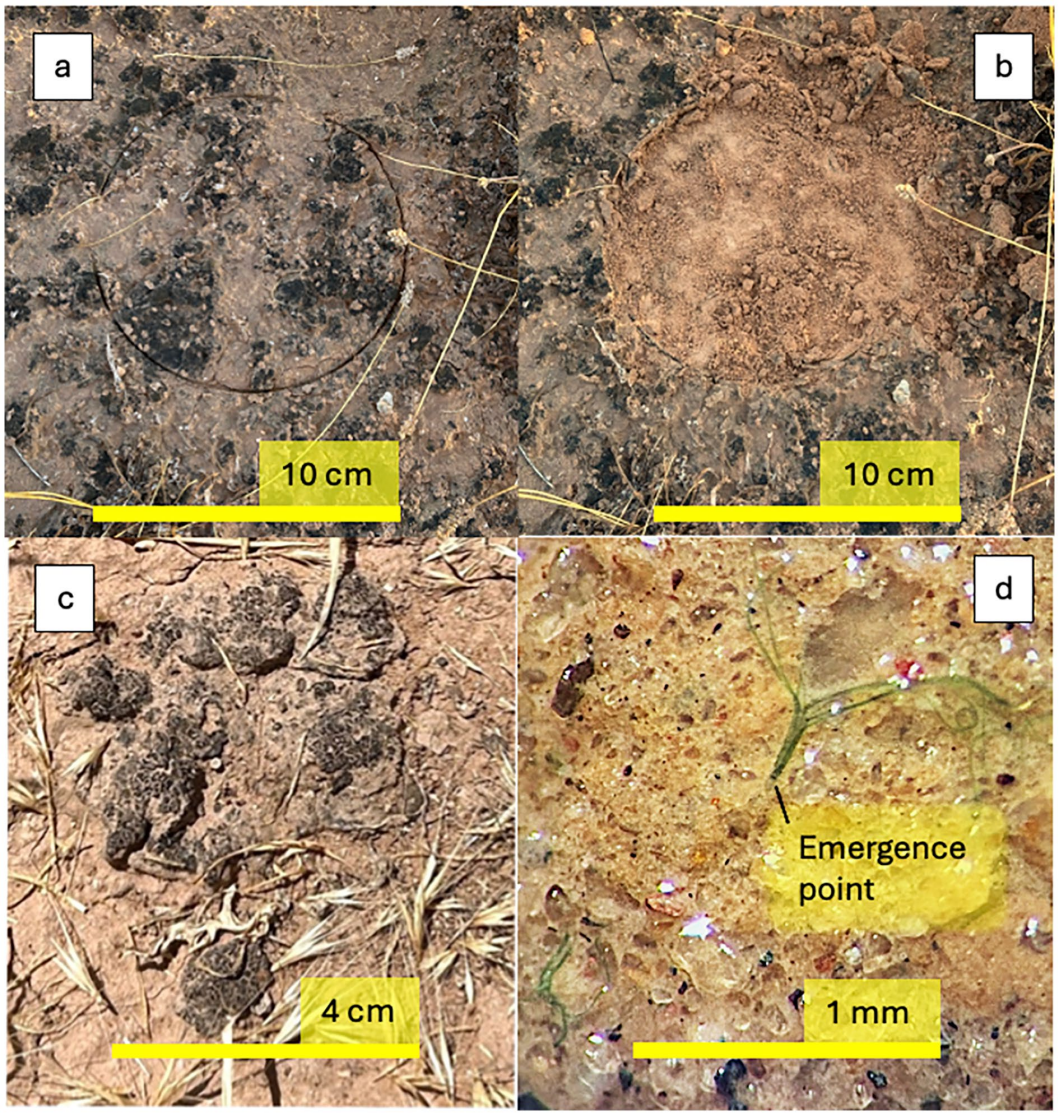
Typical appearance of experimental plots (site ND shown). **(a)** Intact Biocrust before scalping, with a 10 cm diameter demarcation. **(b)** Exposed Undercrust, after scalping plot ND. **(c)** Transplanted whole Biocrust from site ND to crustless farm soil. **(d)** Microscopic detail of Undercrust surface showing emergence of cyanobacterial bundles upon laboratory incubation.

Test plots were marked on crusted soils in December 2023 using 10 cm diameter Petri dishes (Figure 1a). Three plots were set up per site. A secondary (or fourth) plot was set up for re-inoculation at each site. We analyzed three different types of samples in each plot, which we refer to as: (i) “Biocrust,” which constitutes the mature, undisturbed, natural biocrust upper layer, self-defined in thickness by its cohesion, roughly 0.5 cm deep, (ii) “Undercrust,” which is the natural soil that remains exposed after the upper layer is removed, sampled to a depth of 1 cm from the new surface, and (iii) “Recovering,” which corresponds to the incipient microbial community developing with time on the exposed undercrust substrate, sampled to a depth of 1 cm.

Procedurally, and after taking 5 Biocrust internal replicate analytical samples in each main plot, the upper layer (Figure 1a) was carefully removed (Figure 1b), followed immediately by collection of Undercrust analytical samples.

We then inoculated one secondary plot at each site with an equivalent of ~ 5% areal coverage with crumbles obtained from its upper layer (“Re-inoculated”). The main plots received no inoculum. Inoculum preparation involved drying, crushing and sieving, followed by broadcast spreading of the resulting material. One additional inoculation variation involved harvesting and placing, face up, large (2–10 cm) intact biocrust pieces from site ND on the adjacent crustless farm soil (Figure 1c), to assess lateral expansion potential.

Analytical samples (Biocrust, Undercrust, Recovering, Re-inoculated) were collected with a 1 mL Falcon tube to a depth of ~ 1 cm. Five sub-samples from each of the three scalped plots were thoroughly mixed and consolidated into a single composite sample for downstream analyses. The experiment began (plots sampled) on December 28, 2023, and plots were again sampled on May 15, 2024. During this period, there were approximately 17 rainfall events, averaging 6 mm and ranging from 0.5 mm to 25.4 mm.

### 2.3 Laboratory assessment of undercrust cyanobacterial viability and recovery potential

To collect Undercrusts, we pre-wetted each Biocrust and drove a 50 mm diameter Petri plate into the soil. With this method, the upper biocrust layers were lifted, intact, with the Petri plate. We then removed the demarcated upper layer and an additional 50 mm radius to reduce the chances of undercrust contamination from peripheral biocrust aggregates. Then Petri plates were driven into the inner circle of exposed undercrust and its contents sheared laterally over a plate lid or trowel, removed, and allowed to dry. In the lab, samples were re-wetted and inspected under a dissecting scope to confirm the absence of cyanobacterial filaments, which would hydrostatically green-up within 20–40 min (Pringault and Garcia-Pichel, 2004). These were removed, if present, but this was rare. The plates were then subjected to repeated wet/dry cycles: wetted and covered for 3 days (to prevent evaporation), 1 day with the lid partially removed to allow slow drying, then 3 days uncovered (dry), to mimic the natural growth regime in a time-condensed mode. Units were incubated in a light room at ~ 50 μE m^−2^ s^−1^ to allow photosynthesis and encourage migration to the surface (Scott et al., 2025).

### 2.4 Microbial pigment analyses and quantification

Chlorophyll a (chla) was extracted from pre-weighed soil aliquots (~1 g) from each plot and ground with a mortar and pestle in 90% aqueous acetone (Giraldo-Silva et al., 2019a). Acetone soil slurries were centrifuged, vortexed for 30 s, and then stored in the dark at 4 °C for at least 24 h, after which they were vortexed and centrifuged once again. Discarding the soil pellet, the acetone extract was analyzed using a Shimadzu UV-1601 UV–visible spectrophotometer, measuring absorbance spectra from 330 to 800 nm. Chla-specific contributions were deconvoluted and quantified as described in Garcia-Pichel and Castenholz (1991), expressed as mass per area (mg m^−2^) or mass concentrations (mg g^−1^ soil), depending on the data use needs.

### 2.5 DNA extraction and 16S rRNA community composition determination

After pooling plot samples, except at site LV and SF, where plots were analyzed separately (without compositing) the combined soil was gently crushed and mixed in a mortar and pestle. We extracted DNA using a PowerSoil extraction kit (Qiagen) using ~100 – 250 μg of soil following the manufacturer’s instructions. The number of 16S rRNA gene copies was quantified by qPCR (Heredia-Velásquez et al., 2023) using primers 338F (5′-ACTCCTACGGGAGGCAGCAG-3′) and 518R (5′-GTATTACCGCGGCTGCTGG-3′) as a measure of bacterial populations. We used PerfeCTa SYBR Light FastMix Rox (Quantabio) amplified on an ABI ViiA 7 thermocycler (Applied Biosystems). Community DNA was analyzed after next-generation sequencing of 16S rRNA genes. Universal bacterial primers 515F/806R were used to amplify the V4 region of 16S rRNA genes, according to the Earth Microbiome Project. Raw FASTQ files from Illumina sequencing were processed using Qiime2 v2023.5, providing a table of Amplicon Sequence Variants (ASVs), excluding singletons.

Taxonomic assignments were initially made using the Greengenes 13_8 database (DeSantis et al., 2006). The complete ASV table was filtered for cyanobacteria (excluding chloroplasts), which were identified using Cydrasil,^1^ following standard procedures (Roush et al., 2021). Then, using the phylogenetic tree visualization tool (iTol^2^), we created a color progression that represents the distance from the tree root (*Vampirovibrio*), with the *Leptolyngbya* clade being the closest neighbor and the *Tolypothrix* clade being the furthest. The color gradient has an abrupt shift from greens to browns, where browns represent heterocystous cyanobacteria capable of scytonemin production (Scott et al., 2025). The proportion of cyanobacterial reads multiplied by the qPCR-obtained total number of 16S rRNA copies gave the size of the cyanobacterial population. “Other bacteria” are the difference between cyanobacteria and the total.

### 2.6 Calculations and data for figures

All code for calculations and figure generation was prepared in R (R Core Team, 2017). An R-markdown knit file, and raw data, are included in the data repository. We conducted a constrained analysis of principal coordinates (CAP), which is based on a Principal Coordinate Analysis (PCoA) of a distance/dissimilarity matrix, using the vegan package. Vegan was also used to calculated statistical differences in the data, including diversity measures. Both Jaccard and Bray-Curtis dissimilarities were analyzed, constrained by sample *Type* using the *capscale* function. The resulting ordination visualizes variation in community composition attributable to sample type, with ellipses representing 95% confidence intervals for each group. The 16S gene abundance data were visualized with volcano plots using the package edgeR. The log-fold change (logFC) was plotted against the average normalized gene counts [log-counts per million (logCPM)], showing the relationship between the magnitude of differential expression and overall gene abundance between sample *Type* (either Undercrust versus Biocrust, or Undercrust versus Recovering biocrust). All ASVs are shown on the figures, and those whose differential abundance met the stated significance thresholds are highlighted and labeled by genera. Recovery values were calculated according to the equation for resilience described by Yi and Jackson (2021):


$$\frac{\text{Chla}\;(\text{Recovering})-\text{Chla}\;(\text{Undercrust})}{\text{Chla}\;(\text{Biocrust})}$$

**Figure 2:**
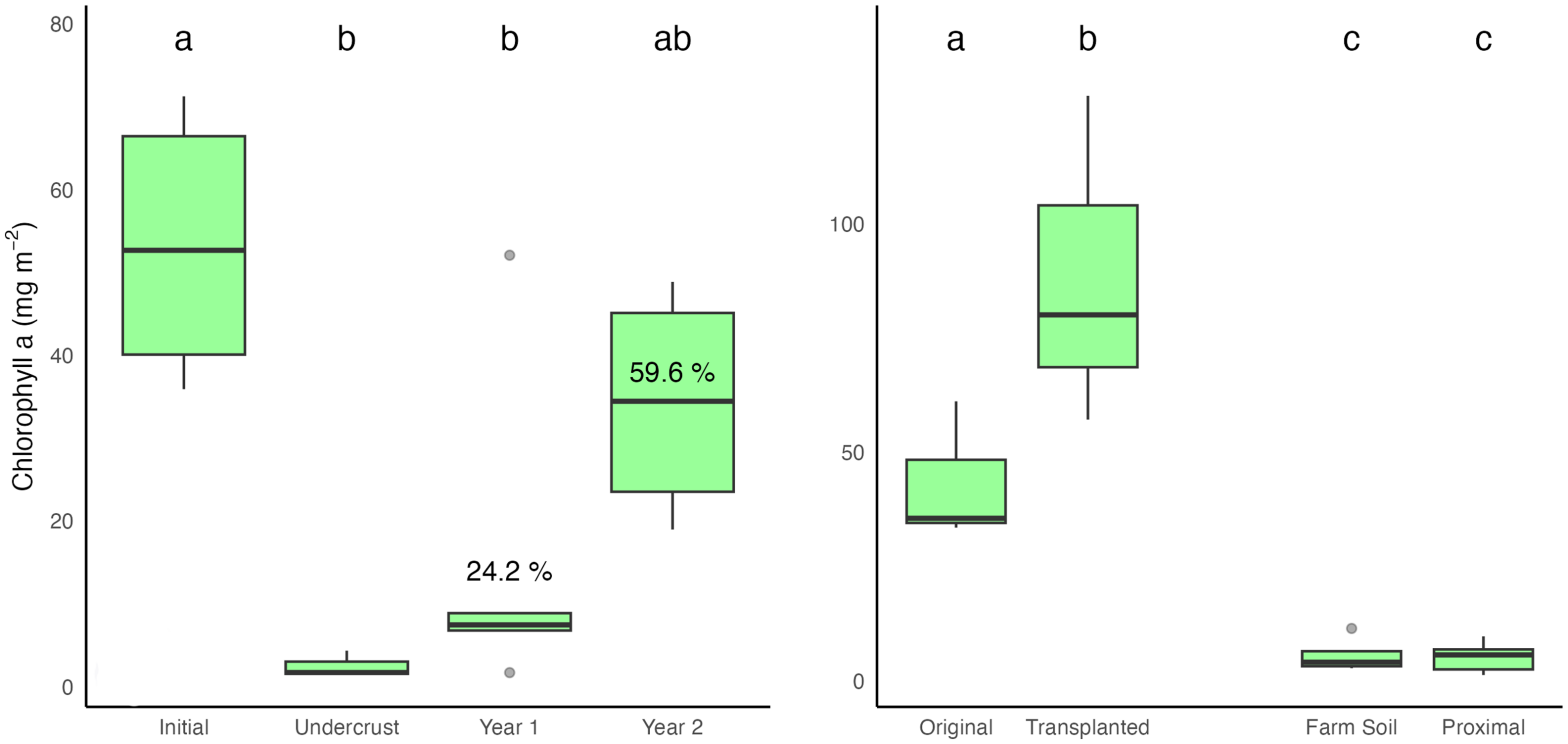
Areal chlorophyll a content during recovery. Statistical differentiation **(a-c)** is based on an ANOVA/Tukey analysis with *p* < 0.05. LEFT: near site DG, after 1 and 2 years of recovery following scalping. Percent recovery (Year 1 – Undercrust)/(Initial) is shown near boxes. RIGHT: Chlorophyll levels in bulk biocrusts transplanted to the farm site (both original and after 4 months of growth), the farm soil itself, and in the area proximal to the transplanted biocrust.

Differences between samples (i.e., Figure 2) were calculated using ANOVA/Tukey.

## 3 Results

### 3.1 Microbial differentiation of upper and undercrust layers

The Biocrust always contained much larger populations of cyanobacteria than the Undercrust (both measured with 16S rRNA copies or with chla content as a proxy), between 2 and 4 orders of magnitude larger based or ribosomal counts, and between 2 and 3 orders of magnitude larger based on chla, as seen in the site-paired ratio shown in Table 1. These vast differences are consistent with previous determinations in the literature (Garcia-Pichel, 2003; Beraldi-Campesi et al., 2009; Xu et al., 2023). The differences were extensive to populations of other bacteria (Table 1), for which site-paired ratios varied between 3- and 200-fold, and thus were somewhat less marked than those found for cyanobacteria.

**Table 1:** Bacterial population densities and chlorophyll a content of Biocrust, Undercrust, and Recovering by site.

Measurement	Layer	SF	DG	LV	DD	ND	LC	25	31
Cyanobacteria ribosomal gene counts	Biocrust	7	103	87	44	72	55	90	139
	Undercrust	0.06	0.74	0.31	0.01	0.02	0.00	0.40	0.28
	Recovering	2.0	20.3	3.9	1.3	0.2	0.3	3.5	2.5
	B: S Ratio	108	138	283	4,323	4,422	67,812	227	503
	% Recovery	28.8	19.0	4.1	2.9	0.3	0.6	3.5	1.6
Other bacteria ribosomal gene counts	Biocrust	13	258	157	203	267	160	278	127
	Undercrust	2.3	61.9	31.1	2.1	1.2	1.0	45.4	48.9
	Recovering	4.72	67.56	16.58	7.49	0.71	2.17	84.81	45.81
	B: S Ratio	5.6	4.2	5.0	95.7	217.1	161.7	6.1	2.6
	% Recovery	18.2	2.2	−9.3	2.7	−0.2	0.7	14.2	−2.5
Chlorophyll a	Biocrust	51.30	91.02	55.18	117.26	49.45	91.89	134.35	89.90
	Undercrust	0.02	2.79	1.39	4.19	0.56	0.02	4.77	0.87
	Recovering	26.7	40.2	20.8	34.2	4.1	18.4	11.6	8.3
	B: S Ratio	414	33	40	28	88	723	28	104
	% Recovery	52	41	35	26	7	20	5	8

Population densities are in units of 10^5^ 16S rRNA gene copies per mg of soil, Chlorophyll a in μg g-soil^−1^. B: S indicates parameter ratios between Biocrust and Undercrust. Recovery (%) for a given parameter was calculated as the difference between recovering and Undercrust values divided by original value found in Biocrusts, multiplied by 100. Measure values represent the average of three replicates samples.

The two layers also differed with respect to community composition based on ribosomal sequence, followed by bioinformatic comparisons. Some patterns were common to all locations: Biocrusts had lower relative bacterial populations of *Actinobacteria* and higher relative populations of *Proteobacteria* and *Bacteroidetes* (Supplementary Figure S2a), which is consistent with a generalized view of their oligo- vs. copiotroph nature, respectively, and their differential response to wetting pulse length (Garcia-Pichel, 2023; Garcia-Pichel and Sala, 2022).

**Figure 3:**
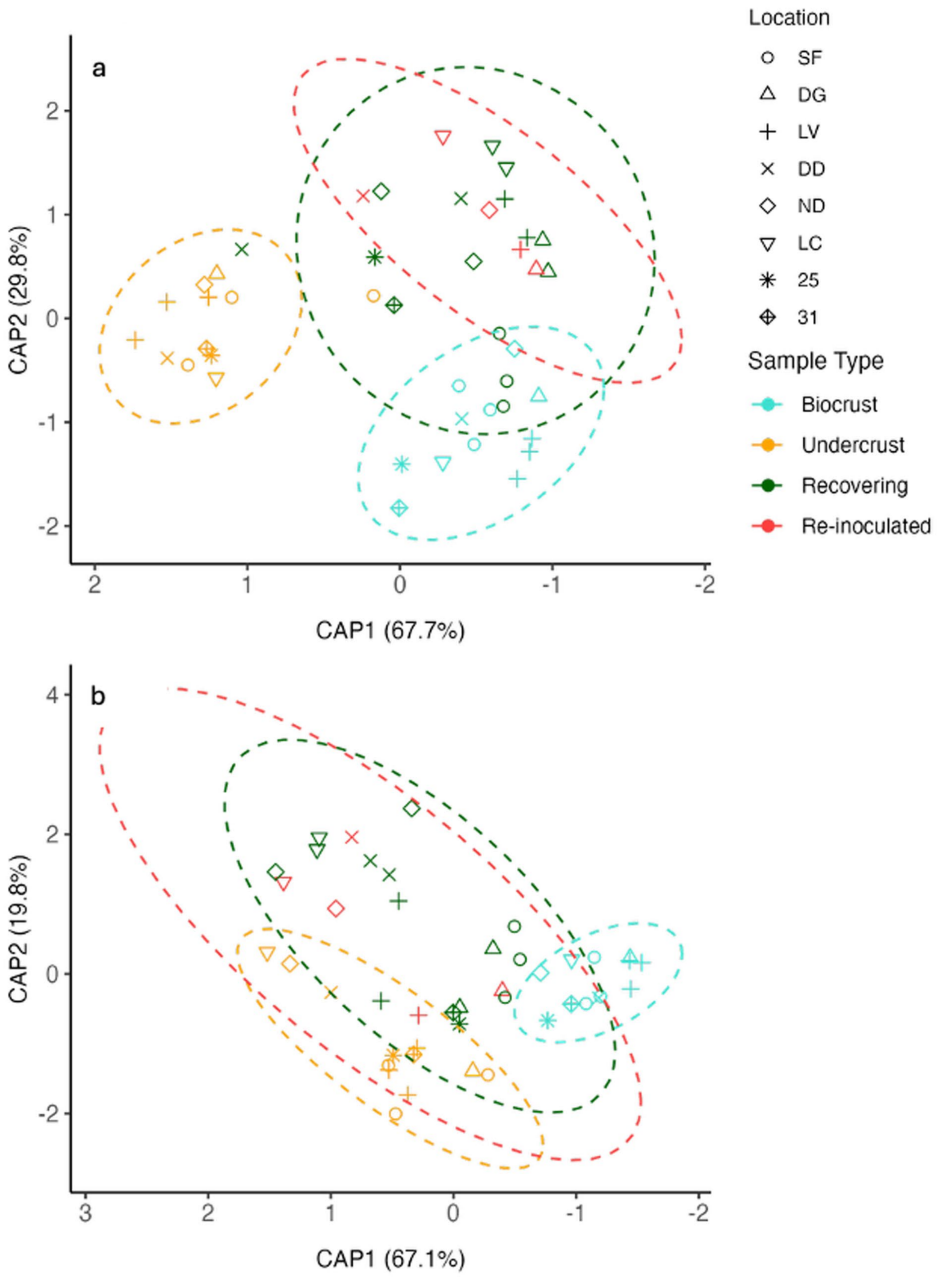
Constrained analysis of principal coordinates (CAP) projection based on a Bray–Curtis dissimilarity matrix for communities of **(A)** cyanobacteria and **(B)** all other bacteria.

There were no patent differences in the relative proportion of cyanobacterial genera across locations, but *Microcoleus* tended to gain relative representation in most Undercrusts (Supplementary Figure S2b). The set of cyanobacterial taxa detected in both communities was common, and not unlike what has been found in prior studies as a typical Sonoran biocrust assemblage (Scott et al., 2025; Fernandes et al., 2021; López-Cortés et al., 2001). Using maximal phylogenetic resolution (i.e., based on all detectable Amplicon Sequence Variants, ASVs), we could confirm that communities of both cyanobacteria and other bacteria were statistically different through pairwise multilevel comparisons, applied to both Jaccard (cyanobacteria *p* = 0.002; bacteria *p* = 0.023) and Bray-Curtis (cyanobacteria *p* = 0.001; bacteria *p* = 0.001) similarity algorithms (Supplementary Table S3). This can be visualized in the CAP projection (Figure 3), where all undercrust communities tended to be self-similar and differentiated in composition from those in the Biocrusts, for both cyanobacteria and other bacteria, regardless of site. While Figure 3 is based on Bray–Curtis similarity indices (which factor in both presence/absence and relative proportions of each ASV), similar conclusions can be drawn from analyses based on Jaccard (presence/absence) indices (Supplementary Figure S3).

We tabulated the density of cyanobacterial 16S gene copies in the Biocrust, the Undercurst, and the Farm soil in Supplementary Table S4. We used the average Biocrust and Undercrust values from locations ND and LV since they were proximal to, and had the same soil type, as the fallow farm. As expected, the Biocrust layer had orders of magnitude more cyanobacteria than the Undercrust, but the undercrusts still carried as many as 1.6 × 10^4^ cyanobacterial 16S rRNA gene copies per g soil Yet fewer cyanobacterial copies were detected in the farm soil (2.5 × 10^3^), but to properly interpret the data, one must pair this with observed growth. We saw no evidence of cyanobacterial growth in the lab with farm soil, even when incubated under ideal conditions for an extended period. Therefore, we speculate that most of the DNA from the farm soil are relics from dead cells (Carini et al., 2017).

**Figure 4:**
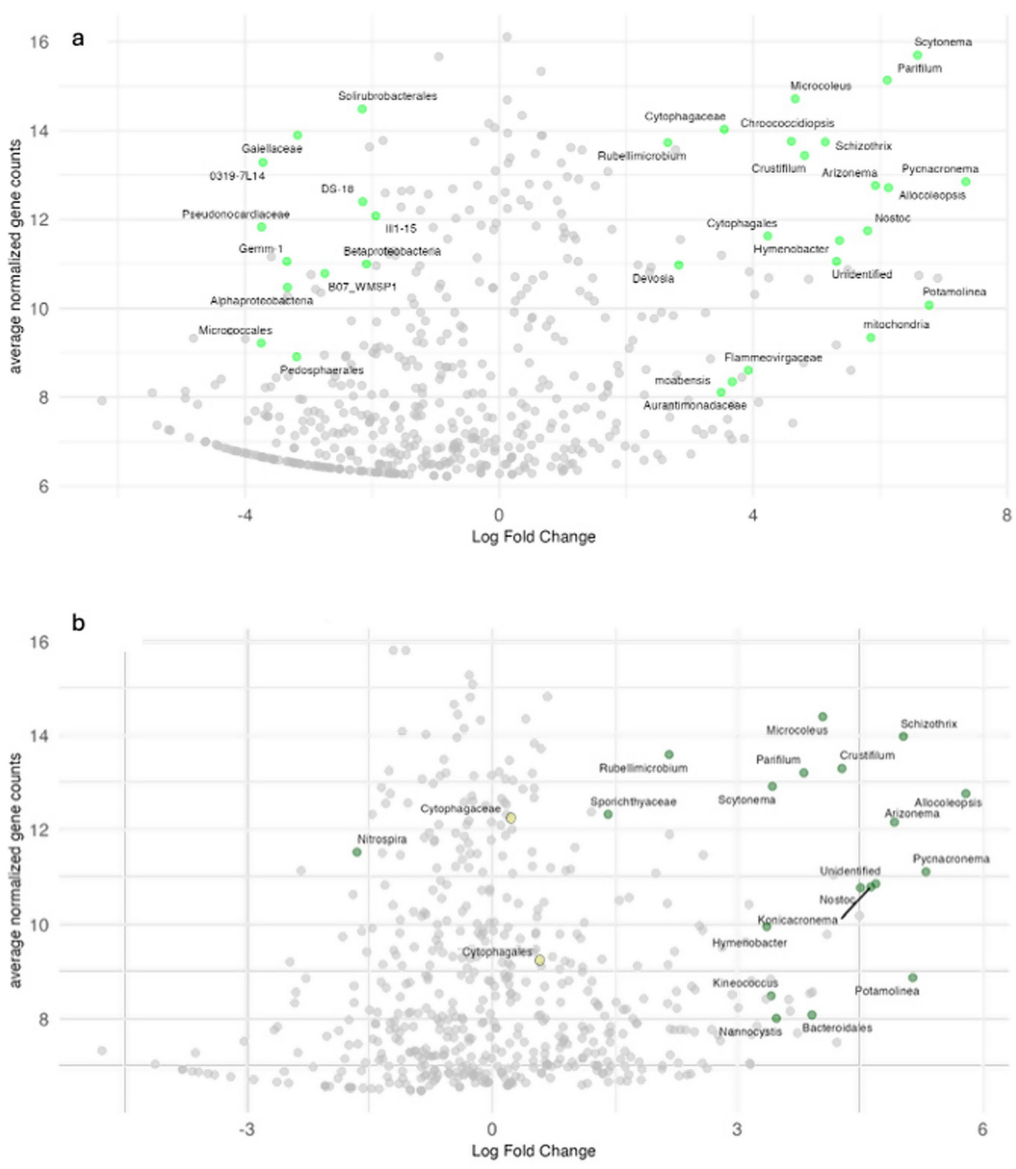
Volcano plots based on EdgeR analyses, comparing ASVs differentially abundant in Undercrusts versus Biocrust **(a)** and Undercrust versus Recovering **(b)**. Colored ASVs (labelled with the best possible taxonomic assignment) had *p* < 0.0005 **(a)** and *p* < 0.01 **(b)**.

To ascertain which bacterial phylotypes were responsible for the community differences detected, we conducted a differential abundance analysis, which are displayed as “volcano plots” (Figure 4). Expectedly, from the results in Table 1, phylotypes that were comparatively (and significantly) more prominent in the Biocrust (Figure 4a) were largely cyanobacteria (right-most in the plot), but with variations among phylotypes (varying by 4 orders of magnitude in differential abundance). For example, *Scytonema* sp. and *Nostoc* sp. among the heterocystous, and *Pycnacronema* sp., *Parifilum* sp., *Arizonema* sp., and *Potamolinea* sp. in the family Coleofasciculaceae (Fernandes et al., 2021) lost much more representation in the Undercrust than *Microcoleus (vaginatus)* or *Chroococcidiopsis* did. Also predictably, phylotypes preferentially and strongly enriched in the undercrust all belong to ‘other bacteria’, and particularly to Actinobacteria (i.e., those assigned to the families *Pseudonocardiaceae, Gaiellaceae, Solirubrobacteriaceae*, and *Micrococcaceae*). In the Recovering biocrusts (Figure 4b), it was largely the cyanobacteria that had recovered, in similar proportions.

### 3.2 Characterization of recovery on exposed undercrusts

We quantified recovery at 5 months following removal of the upper layer by monitoring populations that developed in the now-exposed undercrust through ribosomal gene counts and chla.

Judging from chla, the largest recovery (57%) was at the solar farm (SF, Table 1). Solar farms afford a milder environment, leading to enhanced recovery (Heredia-Velásquez et al., 2023), so one can consider this an upper boundary. All other sites showed some level of recovery, ranging from 7 to 43%. At the site of our original observations (DG), which preceded the survey of sites by 1 year, we monitored recovery for two consecutive years, after which chla levels there had recovered to 24.2% after 1 year and 59.6% after 2 years, statistically equivalent to those in their original, “natural,” biocrusts (Figure 2A).

The recovery of cyanobacterial 16S gene copies was again highest at the solar farm site (29%), varying from 0.3–19% at other locations, and consistently lagging recovery measures by chla, sometimes by as much as an order of magnitude (Table 1). The populations of other bacteria lagged behind those of cyanobacteria in recovery and also had the highest recovery at the solar farm (18%) but failed to recover or even declined at some sites. Re-inoculation with site-specific inoculants, immediately after removal of the upper layer, did not enhance recovery across sites after 5 months (Supplementary Table S1).

The recovery process also involved shifts in community composition according to CAP projections. Recovering cyanobacterial communities differed from those of Undercrusts (Figure 3a, *P* = 0.001), in a spatial placement consistent with a progression from typical Undercrust to typical Biocrust communities. Recovering communities were no longer different in cyanobacterial composition from those in the Biocrusts (Jaccard or Bray-Curtis, *p* > 0.8, Supplementary Table S3). By contrast, “other bacteria” in Recovering communities did not significantly differentiate from those initially in the Undercrusts (Jaccard or Bray-Curtis, *p* > 0.6) and remained distinguishable from those in the Biocrusts (Jaccard or Bray-Curtis, *p* < 0.03), although they crept in placement toward the latter. Re-inoculation did not have an effect, as community composition in inoculated (Re-inoculated) and uninoculated (Recovering) samples could not be distinguished (Jaccard or Bray-Curtis, cyanobacteria or other bacteria, *p* > 0.7). The process of recovery involved massive increases (between thousand and a million-fold) in mostly cyanobacterial ASV’s, of a diverse nature (Figure 4; Supplementary Table S2). No other bacterial ASV of any kind, save for *Nitrospira* (likely a nitrifying bacterium), experienced significant relative losses. This may indicate that disturbances did not necessarily impose adverse conditions on the original undercrust’s “other bacteria” community. Instead, they remained rather impervious to it, both in terms of specific ASV and overall community composition.

### 3.3 The origin of the recovering populations

In the previous section, we demonstrated the swift recovery of biocrusts on exposed undercrust and that re-inoculation made no difference to recovery, neither in composition nor population size. A parsimonious mechanism consistent with those findings calls for the admittedly small, “lost” cyanobacterial populations, residing in the undercrust, to be the source of new growth. This however would imply that they must have been viable. To test this, we set up viability experiments in the laboratory, incubating undercrusts excised from each site under moderate light and recurrent wet/drying cycles in an indoor room. We monitored the undercrusts with a dissecting scope for the appearance of cyanobacterial growth, which eventually occurred in all, although with varying speed. No filaments were observed after two wet/dry cycles. After the third wetting cycle, filaments became visible in DD undercrusts (Table 2; Figure 1d). By the 4th wet/dry cycle, all samples had visible filaments or bundles, which were then enumerated (Table 2).

**Table 2:** Emergence dynamics of cyanobacterial filaments or bundles from underscrusts incubated under wet/dry cycles.

Time wet (days)	Wet/dry cycles	DG	LV	DD	ND	LC	25	31
4	1	0	0	0	0	0	0	0
8	2	0	0	0	0	0	0	0
10	3	0	0	+	0	0	0	0
12	3	0	+	+	+	+	0	0
14	3	+	+	+	+	+	+	+
15	4	10	3.8	14	4.6	3.1	1.5	0.9

Positive indicates where filaments were first detected but were not enumerated. See Figure 1d for a visual companion. Units are filaments per cm^2^.

**Figure 5:**
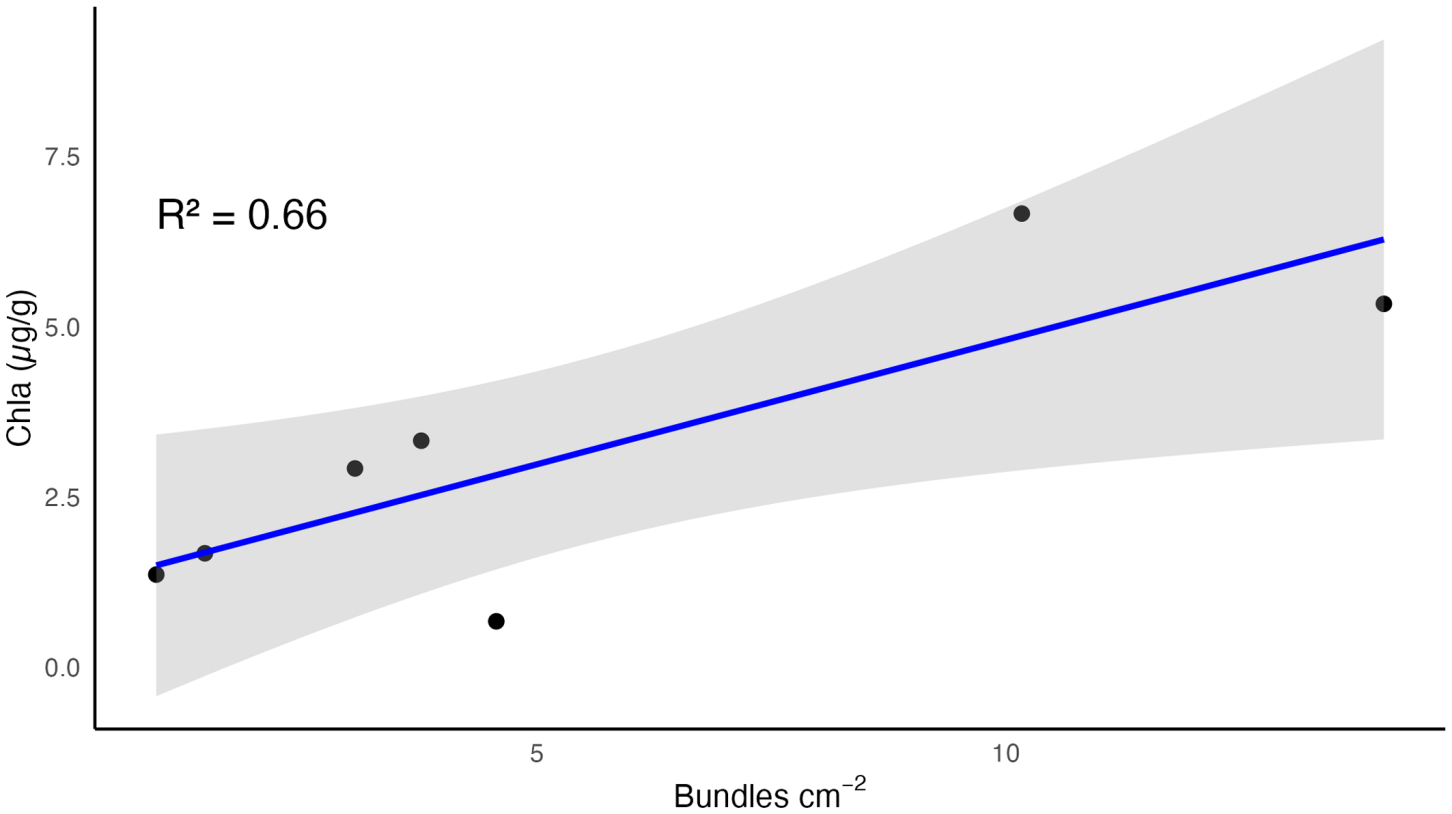
Relationship between areal density of cyanobacterial filaments determined in lab incubations and the level of recovery by end-point chla (recovering).

All enumerated cyanobacteria were bundle formers. *Nostoc* colonies were also present, but they are difficult to distinguish and harder yet to count with confidence. *Scytonema* (and other dark filaments) were rare but also present. This demonstrates that at least some cyanobacteria in the undercrust remain viable and can readily grow, when hydrated and exposed to light. Further, the end-point areal density of filaments/bundles determined in the lab predicted quite well the level of recovery attained in the field experiments by chla (*R*^2^ = 0.66, *p* = 0.03; Figure 5).

### 3.4 Potential for lateral expansion

To assess the potential for lateral expansion, we transplanted large, intact, biocrust pieces (2–10 cm across) from site ND to the fallowed farmed site, where they resided on, and were surrounded by, crustless soils (Figure 1c). At the end of the study period, the area of soil surface surrounding the transplant, and the biocrusts themselves, were monitored for changes in cyanobacterial populations through chla analyses. Biocrusts survived transplantation and saw an increase in chlorophyll a (Figure 2B). However, soils ca. 2 cm around the transplants were no different from the background levels in farm soil indicating that lateral propagation had not occurred.

## 4 Discussion

### 4.1 Widespread resilience from down under

Our experiments involving scalped Biocrusts established that recovery was generally fast, and hence that under this scenario, they are resilient communities, with the potential for significant recovery in terms of cyanobacterial biomass and community composition within a single season. Resilience in the face of such extreme interventions may come as a surprise given the widespread view that biocrusts recover very slowly. Nevertheless, the literature contains examples of both relatively fast and slow recoveries, which has also been our experience. When we removed the upper layer from a 200 m^2^ area near location DG, we detected a 31% recovery through the 2022/2023 winter rainy season lasting only several months (Supplementary Figure S4), which is consistent with the data reported here (Table 1). Other studies where existing biocrusts were “scalped” from the surface tend to show significant recovery within similar time-frames in uninoculated plots (Faist et al., 2020; Román et al., 2021; Callison et al., 1985; Cole and Brooks, 2000; Antoninka et al., 2020; Bowker et al., 2020; St. Clair et al., 1986). For example, Rubio and Lázaro measured some 15% recovery in the Tabernas Desert (Spain) (Rubio and Lazaro, 2023), Dojani 10% in the South African Karoo (Dojani et al., 2011), Jech some 13% in the US Colorado Plateau (Jech et al., 2023), and Hawkes reported 11% in Florida shrublands (Hawkes and Flechtner, 2002). Similarly, when destroyed by fire, recovery was also quick (Callison et al., 1985). In contrast, studies where the experimental intervention must have precluded the preservation of remnant undercrust, often report no recovery in inoculated plots (Eldridge and Ferris, 1999; Chiquoine et al., 2016; Fick et al., 2020; Chock et al., 2019; Zhao et al., 2021), or only long-term recovery (Schultz et al., 2022). These three apparent patterns: (1) speedy recovery with an extant undercrust, (2) recovery in control plots with an extant undercrust, and (3) lack of recovery without an undercrust, all point to the undercrust communities as essential players in biocrust resilience.

Undercrust layers host diverse cyanobacteria, but of very low population density (Table 1). Most members were genetically identifiable as typical biocrust inhabitants that were also present in the upper biocrust layer. They could thus principally constitute a biodiversity reservoir, or seed bank, responsible for high resilience. An examination of microbiome changes during recovery seems to support this notion, as many cyanobacterial phylotypes preexisting in the undercrust quickly grew, consistent with the view that cyanobacteria are Nimble Responders (Garcia-Pichel and Sala, 2022; Kut and Garcia-Pichel, 2024; Young et al., 2025) (Figure 4). The level of Shannon diversity observed in the Biocrusts (2.9 ± 0.1, Supplementary Table S2) was fully restored in the Recovering samples (3.0 ± 0.1), compared to that found in the Undercrusts (2.2 ± 0.2). Most disturbed soils, which generally experience a loss of diversity, take much longer to recover in composition and function (Moreno-Mateos et al., 2017). From a community composition standpoint, cyanobacteria in the Recovering soils, after one winter rainy season, had become statistically indistinguishable from communities of mature Biocrusts (*p* = 0.8, Supplementary Table S3). We speculate that cyanobacteria in the newly exposed undercrusts may have benefited from Negative Frequency Dependence (Wisnoski and Lennon, 2021), where, once exposed, any viable cyanobacteria now in the surface can grow unfettered without much competition for resources (Philippot et al., 2021), much like an algal bloom, and with fewer antagonists (Shiah et al., 2022).

For “other bacteria,” any community composition shifts during recovery were not significant (Figure 3b; *p* = 0.60, Supplementary Table S3), although they may have been incipient (Figure 3b) as if lagging behind those of the cyanobacteria, on which they were metabolically dependent. Concerning ecological diversity, and consistent with observations that cyanobacteria exert a filtering effect on heterotrophic diversity (Maier et al., 2018; Lee et al., 2016), Shannon indices of Recovering samples did not differ from those of the Undercrusts (6.34 ± 0.05 vs. 6.38 ± 0.06; Supplementary Table S2). Both were somewhat more diverse than the Biocrusts (6.14 ± 0.03), suggesting that filtering by interactions with cyanobacteria had yet to take effect.

### 4.2 Survival of undercrust cyanobacteria

That the undercrust cyanobacteria led the charge in recovery could be challenged on the grounds that light does not penetrate deep enough into the soil to support their photoautotrophic metabolism because the euphotic zone extinguishes in less than a mm (Garcia-Pichel et al., 2003). Cyanobacteria could not possibly act as primary producers in the Undercrusts. Still, a few cells or filaments could be carried downwards from the surface layer with percolating precipitation, or, for motile forms, could stray in their migrations, but only a very small proportion of individuals would be subject to such fate. Specific differences among cyanobacteria regarding how prone they are to stray or be transported into deep areas and specific differences in their ability to remain viable in the dark likely contribute to the community differences detected. These mechanisms must also affect other bacteria (largely heterotrophs), but in this case, emigrating bacteria (for example, copiotrophic Proteobacteria) merge with local, preexisting soil populations at depth (i.e., enriched in oligotrophic Actinobacteria). The direct measures of viability of undercrust cyanobacteria presented in Table 2 support such conjectures, even if not proving or fully explaining them.

Dryland cyanobacteria and other bacteria have evolved high desiccation tolerance (Soule and Garcia-Pichel, 2019; Marks et al., 2025), a condition that prevents chemical degradation of cellular structures (Bosch et al., 2021). Dried crushed biocrusts or dried cyanobacteria cultures can survive storage for extended periods in the dark (Chiquoine et al., 2016; Giraldo-Silva et al., 2019b) and serve as foolproof inoculum in the lab and greenhouse settings. But dry undercrusts will be moistened during rainfall events as small as 3 mm, which are quite common. Re-hydrated cyanobacteria are subject to cellular decay if physiological repair cannot be powered in some way (Imminger et al., 2024). While many, but not all, cyanobacteria display moderate heterotrophic potential (Soule and Garcia-Pichel, 2019; Stebegg et al., 2023), this is typically insufficient for competition for organic carbon as a source of energy with true heterotrophs, but perhaps sufficient to sustain viability in the dark, particularly because photodamage (Raanan et al., 2016) will be negligible in the undercrust. The precise physiological mechanisms for maintenance of such long-term viability in soil cyanobacteria seem worthy of additional study.

### 4.3 Predictive power of undercrust cyanobacteria

Lab-incubated undercrusts showed evidence of viable cyanobacteria that varied in density by approximately one order of magnitude (Table 2). Similarly, recovery level in the field based on chla levels also varied by approximately one order of magnitude (Table 1). Comparing these values provides the compelling correlation (Figure 5) that the areal density of viable cyanobacteria from the undercrust could predict the eventual recovery in the field. A logical question to ask is why undercrusts do better than interventionally supplied local inoculum, when they demonstrably consist of genetically similar species with the same origin. One obvious difference is that the resident undercrust microbes are embedded in a structured soil matrix (Figure 1c) and are able to take advantage of its alleged refugia, whereas those supplied as crumbled or slurried preparations cannot. Taken as a whole, the evidence suggests that the undercrust indeed provides a structured refugium (Rillig et al., 2017; Baho et al., 2012) for a viable seed bank responsible for resilience.

### 4.4 The dependence of recovery mechanisms on severity and type of disturbance

The level of disturbance would clearly play a role in biocrust recovery. With a *de minimis* disturbance, such as mild compaction, biocrusts are not appreciably affected and would not need to recover. Under mild trampling (Kuske et al., 2012), some degree of recovery may be needed, particularly if lateral movement introduces soil shearing that disrupts aggregates and pore structure. In a disturbance with severe loss of the upper layer there may be microorganismal migrations from the periphery, as demonstrated in greenhouse experiments (Sorochkina et al., 2018), or abiotic input of propagules through dust deposition, or carried out by overland flow. We imposed an extreme disturbance (scalping), which maximizes the relative significance of the undercrust seed bank. However, such disturbances are observed in natural systems and may occur due to trampling (Supplementary Figure S4) or in folded biocrusts (Beraldi-Campesi et al., 2014) (Supplementary Figure S5). Thus, how much the seed bank contributes to a given recovery may vary.

A case has been put forth that restoration by interventional inoculation may be futile given the ubiquity of biocrust distributions, the global dust transport patterns, and the presence of airborne microbes in the dust, which together guarantee that an appropriate inoculum will swiftly reach crustless areas (Warren et al., 2019). However, the claim relied on reasoned inference only. Depositional recovery seems very unlikely for our area and study period, since we found no evidence of populational “founder effects,” neither in the recovering communities, which quickly reached high diversity (Supplementary Table S2), nor in the variability between communities among sites that one would expect in such a stochastic form of recovery. In prior studies from our group, it took 6 months to find natural depositional inoculation of test soils by one cyanobacterial species and an additional 4 months to detect a few more, which denotes clear founder effects (Sorochkina et al., 2018). To explain our current results on this mechanism, one would have to postulate that dust deposition was so common and effective as to abolish such stochasticity. And yet, during our experiments, there were no dust storms or even intense winds. Rather, recurrent winter rains kept soils moist, curbing the local formation of fugitive dust. Further, yearlong microbial surveys of airborne dust carried out in the same area of investigation failed to detect biocrust microorganisms, while detecting a variety of vegetation and human activities in this mixed-use landscape, even rare pathogens, on the basis of microbial signatures (Finn et al., 2021). This is unsurprising given that the microbes in biocrusts are naturally selected to resist wind entrainment. Finally, if airborne inoculum sources were so effective, one should have seen prompt recovery in fallowed farms, which was clearly not the case.

Lateral growth, or lateral migration from the plot edge, could have played a role, particularly since our experimental plots were small (78.5 cm^2^), and so could particulate transport from surrounding areas driven by local forces such as runoff. We did not see this as a major recovery driver for two reasons. Our evidence (admittedly not exhaustive) from control transplants (Figure 1c) failed to detect lateral expansion. Separately, Jech et al. (2023) found no difference in recovery based on plot size ranging from 100 cm^2^ to 10,000 cm^2^ (i.e., no “edge effects”) and did not observe progressive recovery from the edges toward the center.

### 4.5 Implications for biocrust restoration

The practice of setting up experimental plots by scalping the existing biocrust layer is very common in biocrust research, both for basic ecological and restoration purposes. Restoration is often attempted by using inoculants obtained from natural areas (Tucker et al., 2020), grown in greenhouses (Ayuso et al., 2020), or from laboratory-grown primary producers with or without growth adjuvant isolates (Nelson, 2021) to speed natural processes of propagule re-seeding. However, inoculants commonly fail to survive transplantation (Antoninka et al., 2020; Jech et al., 2025). The effective role of the undercrust as a seed bank, shown here, should make practitioners re-evaluate the scalping approach, since it will likely diminish the experimental power of discrimination between treatment effects and controls. Untreated controls, we know now, may show swift recovery, so that treatment effects may easily go undetected. In the results presented here, for example, we could not see the effect of re-inoculation on a fast natural recovery background. Using naturally crustless soils instead, or adding a sterilization (i.e., fumigation) step to protocols after scalping seems advisable. There may also be important conservation and restoration implications, including: (1) biocrust harvesting, for use as inoculum, would not necessarily cause permanent harm to the natural environment, provided the undercrust layer were left undisturbed; (2) necessary disturbances, such as construction of a drylands solar farm, may accelerate soil stabilization recovery by a grading plan that protects, to the extent possible, the undercrust; (3) on highly disturbed land, such as a fallowed farm where there is no undercrust, surface inoculation has proven to be ineffective, demanding an alternate strategy taking advantage of the undercrust seed bank by transplanting whole soil “plugs” may lead to an effective alternative for soil restoration.

### 4.6 The undercrust in context

Post-disturbance recovery from seed banks, a concept that originates in plant community ecology (Luzuriaga et al., 2005; Yang et al., 2021), is known to apply across many ecosystems and communities, from marine corals (Doropoulos et al., 2022), to wetland invertebrates (de Oliveira Hoffmann et al., 2023), and sphagnum peatlands (Wang et al., 2020). Further, we see examples of microbial recovery through dormancy (Kuo et al., 2021; Lennon and Jones, 2011; Caporaso et al., 2012). Our findings suggest that phototrophic seed banks, present in dryland soil undercrusts, are yet another example of this broader ecological paradigm, and one that has immediate implications for arid land management. For example, we now recognize that prior study designs and restoration approaches, including ours, included a latent bias by neglecting to take into account undercrusts and their seed banks.

## Data Availability

The datasets presented in this study can be found in online repositories. The names of the repository/repositories and accession number(s) can be found here: https://www.ncbi.nlm.nih.gov/bioproject/PRJNA1282785/ and https://doi.org/10.5281/zenodo.15931093.

## Footnotes

1
https://www.cydrasil.org

2
https://itol.embl.de
