# Supplementary material for: Soil phototroph community resilience comes from down under
Source: Front Microbiol. 2025 Dec 10;16:1689042. doi: 10.3389/fmicb.2025.1689042 (PMC12727951; doi:10.3389/fmicb.2025.1689042)
Supplement: Supplementary file 1 [file Data_Sheet_1.docx]

**Supplementary Figure 1** Locations of research and other relevant sites. Letter identifiers convey no relevant meaning. Number identifiers represent altitude in 100s of feet above mean seal level, 2500 feet ~ 862 m, 3100 feet ~ 945 m, others were ~ 1350 – 1450 feet ~ 410 – 440 m. The data repository includes a kml file with precise locations.

**
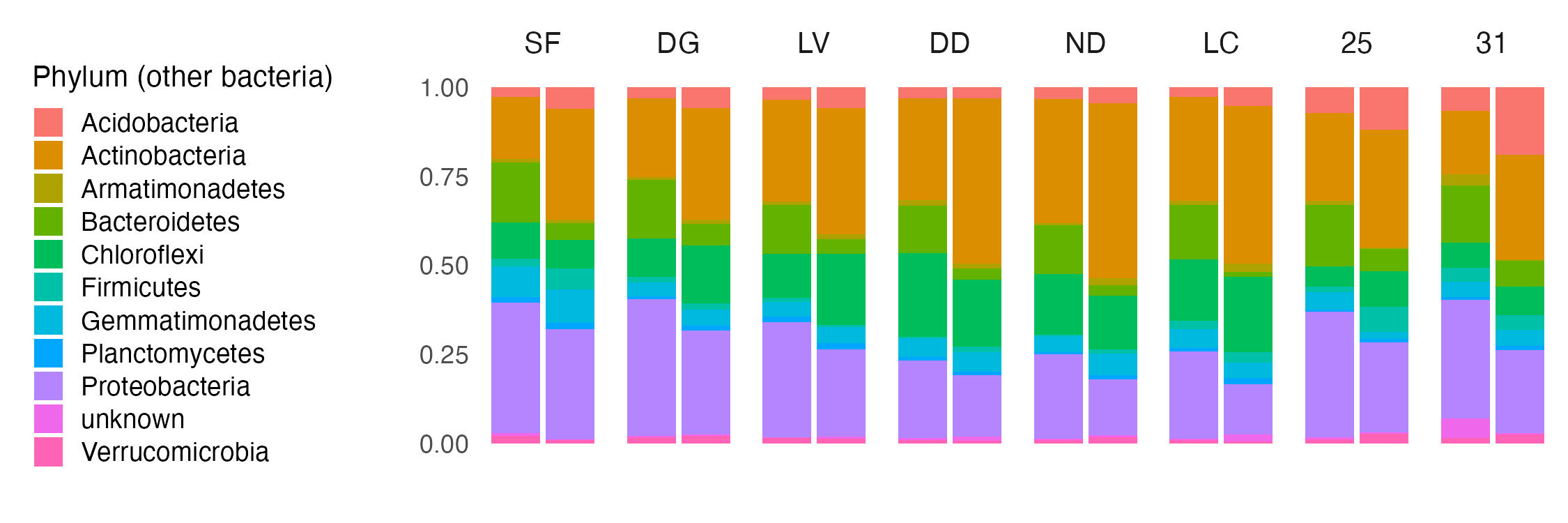
**

b

a


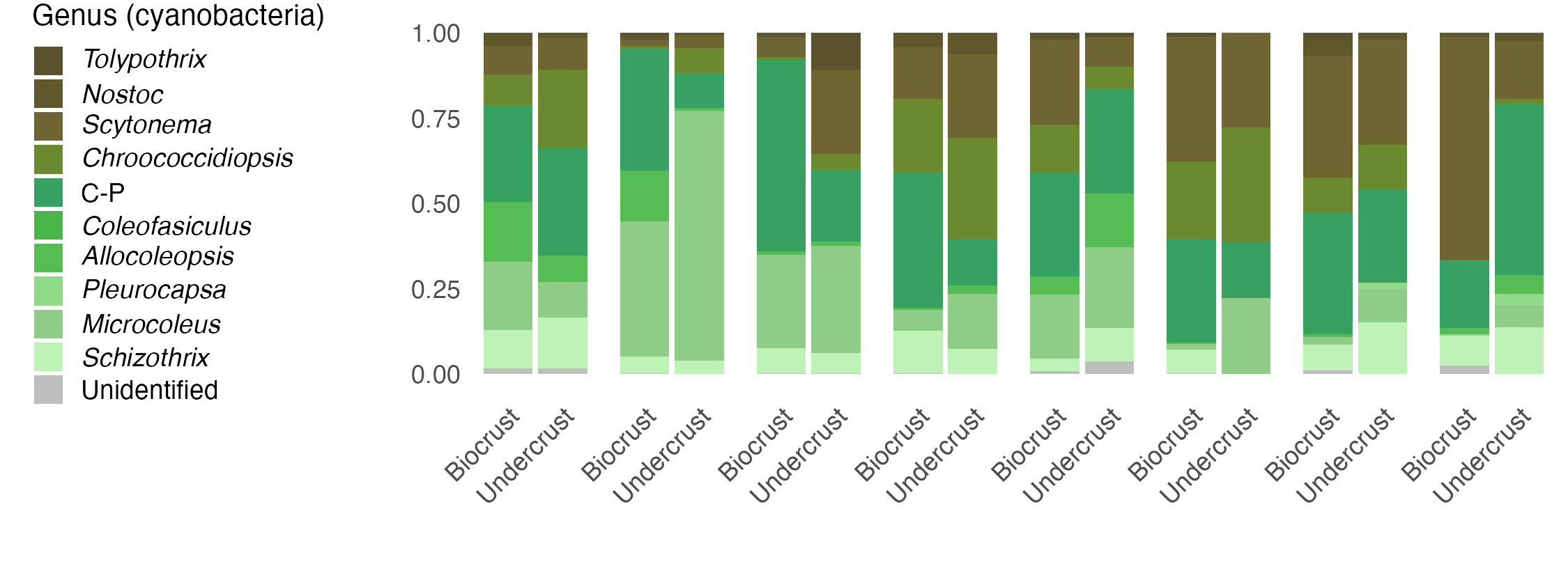


**Supplementary Figure 2** Community composition (relative abundance) of Biocrusts and Undercrusts paired by site. Top chart refers to non-cyanobacterial (other) bacteria, and bottom chart refers to cyanobacteria. Phylogenetic assignment level chosen to highlight differences. For clarity, C-P represents a cyanobacterial clade encompassing Crustifilum, Arizonema, Potamolinea, Pycnacronema and Parifilum. Rare genera (e.g. Konicacronema) were excluded, for visibility.

**
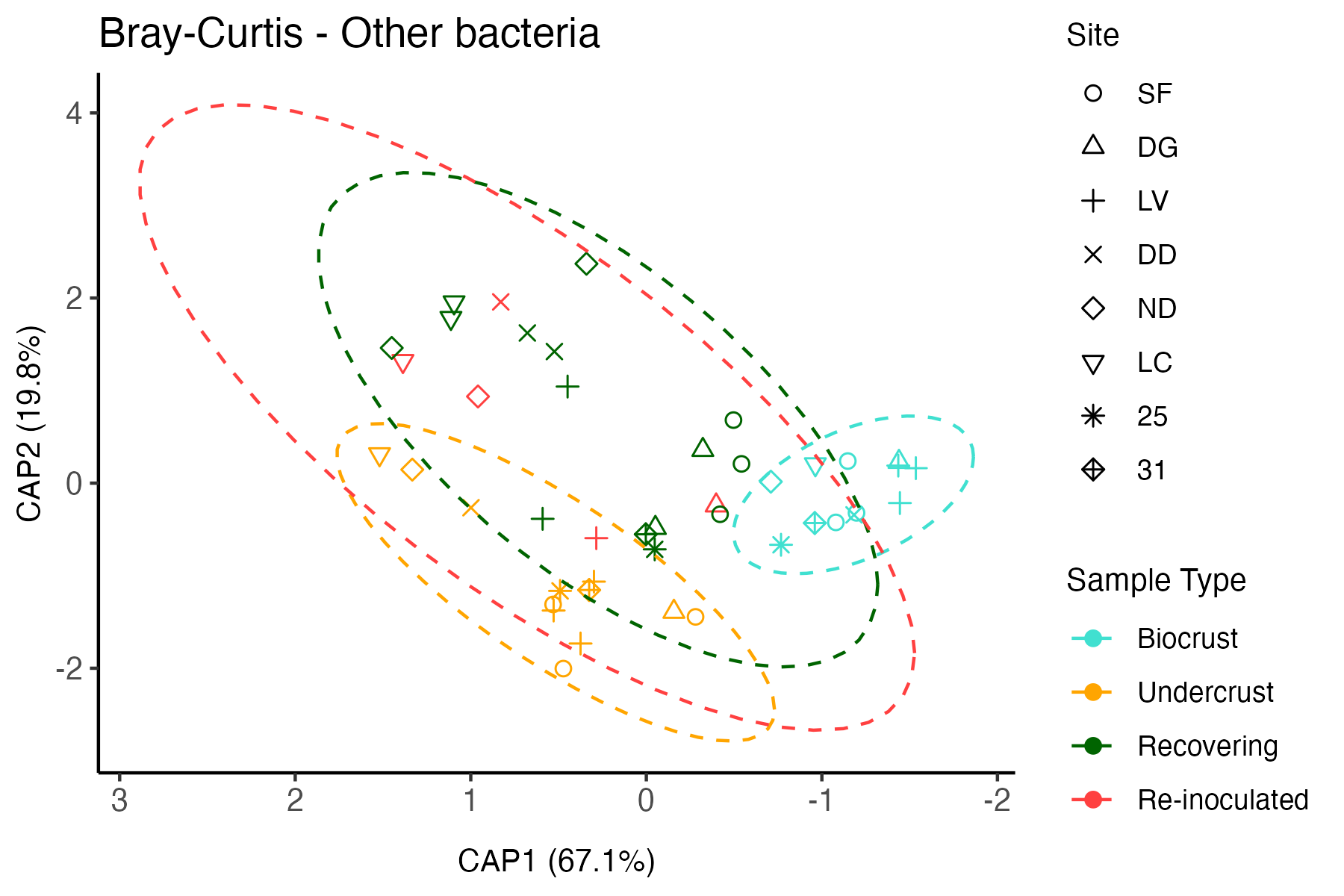

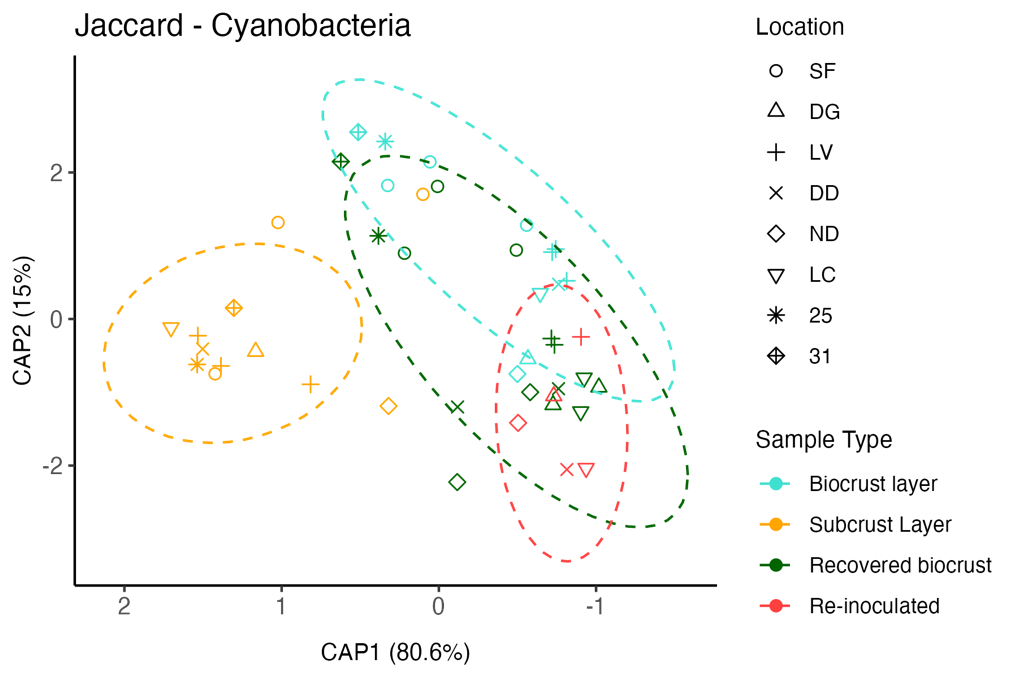

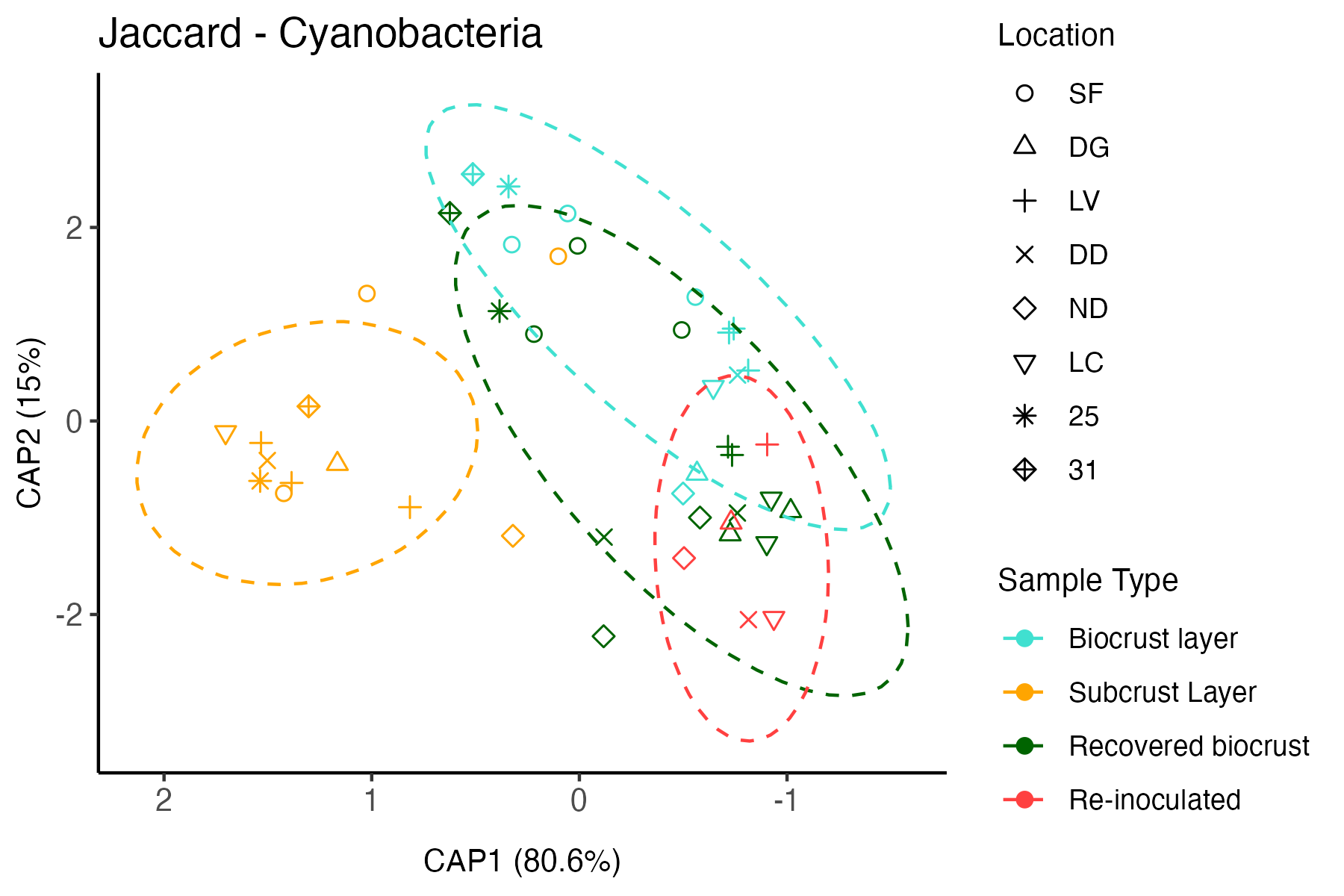

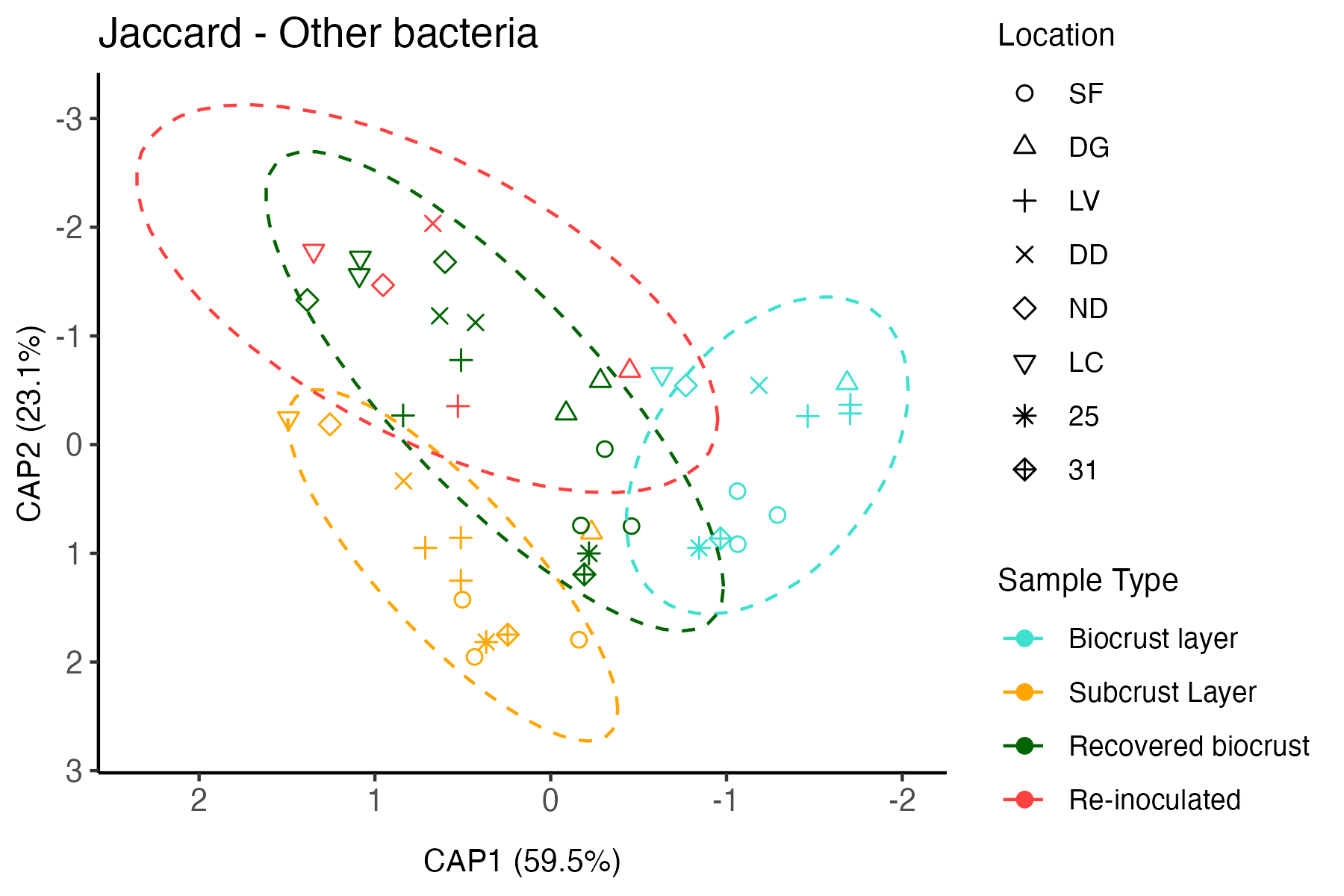
**

a

b

**Supplementary Figure 3** Constrained analysis of principal coordinates (CAP) projection using a Jaccard distance matrix for a) cyanobacteria and b) all other heterotrophic bacteria. CAP
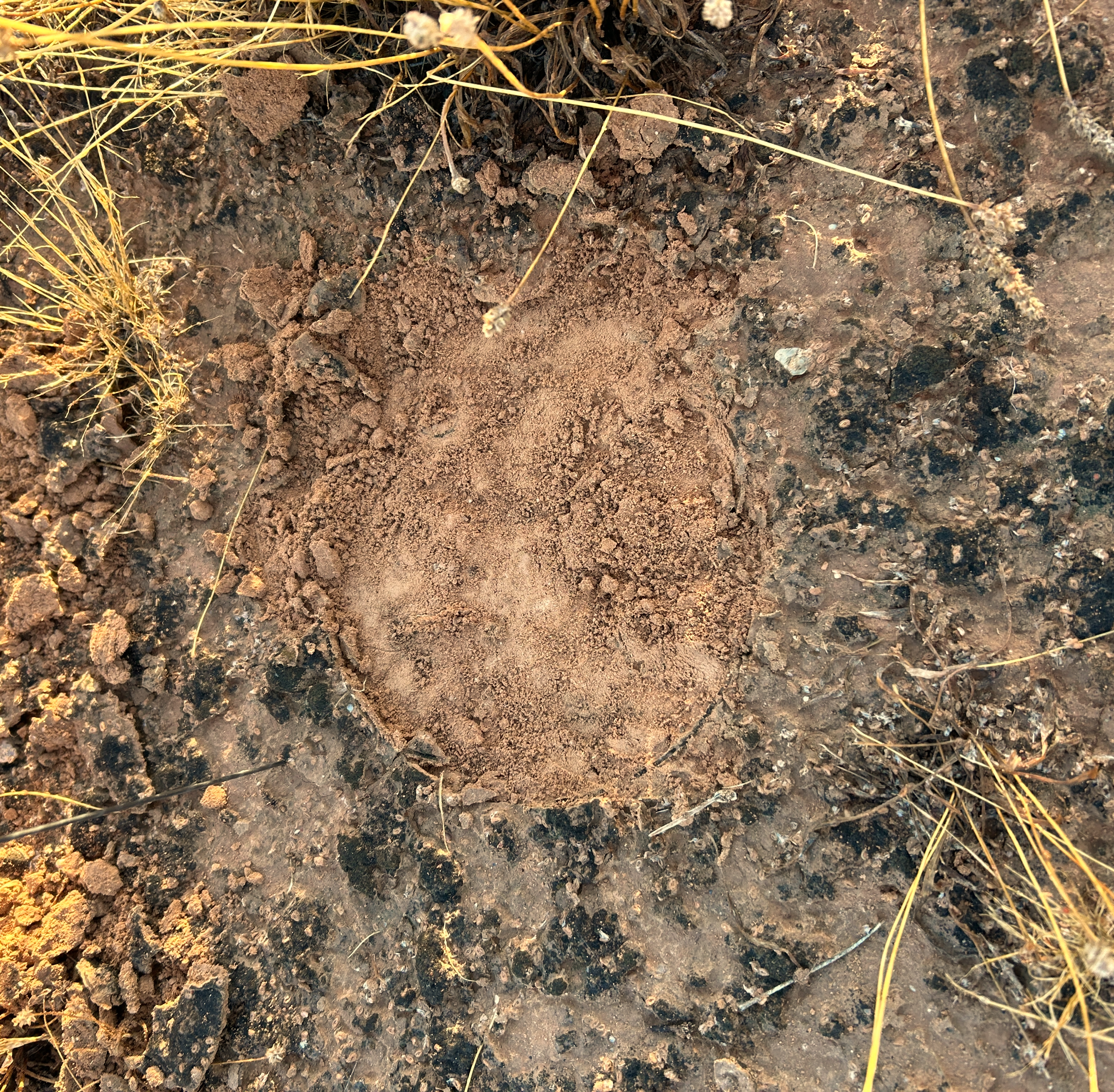
 percentages based on Sample Type.

- Post-harvest


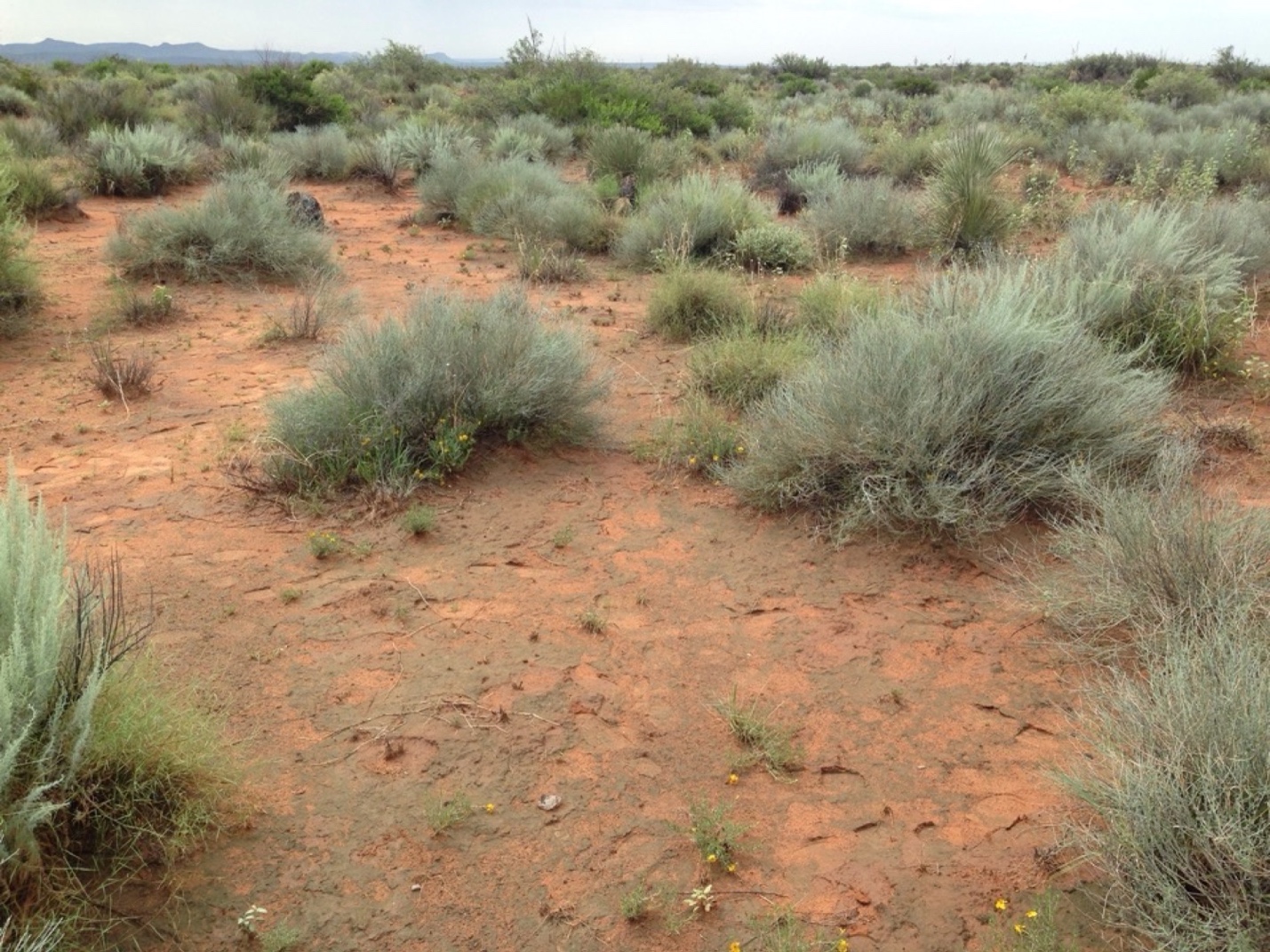


**Supplementary Figure 4** Example site where trampling disturbance in evident, at an aerial scale that is comparable to our test plots. Photo taken at the Jornada Experimental Range near Las Cruces, New Mexico.

**
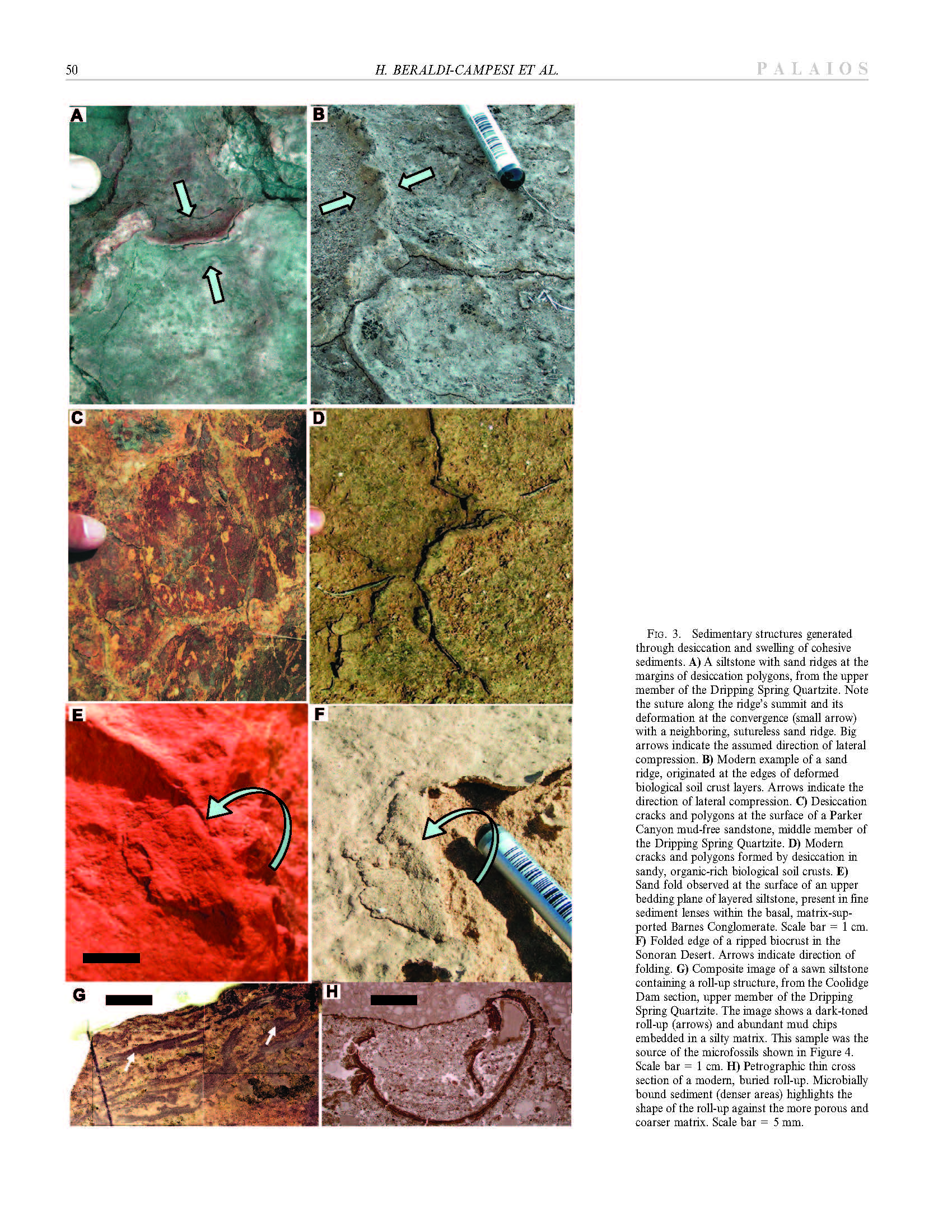
**

**Supplementary Figure 5** Naturally occurring folded biocrust, which is mechanically equivalent to scalping, exposing an area of undercrust. Photo take from (Beraldi-Campesi et al., 2014).

**Supplementary Table 1** Chlorophyll a (mg m^-2^) in uninoculated versus re-inoculated plots by site. Data were not normally distributed, so comparisons are Wilcoxon rank sum test. There was no difference due to re-inoculation (V = 62, p = 0.63).

| Location | Month | Uninoculated | Re-inoculated |
| --- | --- | --- | --- |
| DD | June | 2.64 | 2.41 |
| DD | May | 6.00 | 5.68 |
| DG | June | 2.28 | 4.81 |
| DG | May | 6.63 | 6.53 |
| 25 | June | 1.69 | -0.29 |
| 25 | May | 2.19 | 0.13 |
| 31 | June | 2.25 | 0.81 |
| 31 | May | 1.40 | 0.89 |
| LV | June | 1.41 | 1.30 |
| LV | May | 3.55 | 9.25 |
| LC | June | 0.90 | -0.23 |
| LC | May | 3.16 | 12.50 |
| SF | June | 1.92 | 1.40 |
| SF | May | 4.54 | 7.08 |
| ND | June | 0.16 | 0.11 |
| ND | May | 0.69 | 0.86 |

**Supplementary Table 2** Shannon diversity for communities of cyanobacteria and other bacteria. Values represent average diversity across sites DG, LV, DD, ND, LC, since other sites were not reinoculated. Cyanobacteria in the Undercrusts were less diverse than in Biocrusts or Recovering (inoculated or not) p <= 0.01). For other bacteria, Undercrust were significantly more diverse than Biocrusts (p= 0.07) as were Recovering (p = 0.01). All other comparisons were non-significant (p >0.34). Significance according to ANOVA/Tukey tests. See knit file.

| **Shannon Diversity Index** | | | |
| --- | --- | --- | --- |
| **Type** | **n** | **Cyanobacteria** | **Other bacteria** |
| Biocrust | 7 | 2.9 ± 0.1 | 6.14 ± 0.03 |
| Undercrust | 7 | 2.2 ± 0.2 | 6.34 ± 0.05 |
| Recovering | 10 | 3.0 ± 0.1 | 6.38 ± 0.06 |
| Re-inoculated | 5 | 3.0 ± 0.1 | 6.29 ± 0.07 |

**Supplementary Table 3** PERMANOVA with pairwise comparisons based on Bray-Curtis and Jaccard distance matrices.

|  | Bray-Curtis  P value  Cyanobacteria | Bray-Curtis  P value  Other bacteria | Jaccard  P value  Cyanobacteria | Jaccard  P value  Other bacteria |
| --- | --- | --- | --- | --- |
| Overall  PERMANOVA | 0.001 | 0.001 | 0.002 | 0.023 |
| Pairwise Comparisons |  |  |  |  |
| Biocrust vs Undercrust | 0.002 | 0.006 | 0.002 | 0.005 |
| Biocrust vs Recovering | 0.828 | 0.028 | 0.877 | 0.022 |
| Biocrust vs  Re-inoculated | 0.173 | 0.012 | 0.207 | 0.019 |
| Undercrust vs Recovering | 0.001 | 0.597 | 0.001 | 0.590 |
| Undercrust vs Re-inoculated | 0.001 | 0.427 | 0.001 | 0.514 |
| Recovering vs Re-inoculated | 0.876 | 0.743 | 0.937 | 0.851 |

**Supplementary Table 4** 16S gene copies per g soil at the fallow farm, compared to Undercrust and Biocrust samples from locations ND and LV (taken as an average).

| Genus | Biocrust | Undercrust | Farm |
| --- | --- | --- | --- |
| Allocoleopsis | 128862 | 97 | 83 |
| Arizonema | 321588 | 491 | 0 |
| Calothrix | 41901 | 9 | 0 |
| Chroococcidiopsis | 540338 | 719 | 0 |
| Coleofasiculus | 378 | 0 | 0 |
| Crustifilum | 450265 | 491 | 0 |
| Konicacronema | 27482 | 151 | 358 |
| Lyngbya | 72366 | 72 | 0 |
| Microcoleus | 1873068 | 5020 | 413 |
| Myxacorys | 8204 | 129 | 0 |
| Nostoc | 58090 | 108 | 55 |
| Parifilum | 1991284 | 1673 | 138 |
| Planktothrix | 504 | 108 | 523 |
| Porphyrosiphon | 3656 | 0 | 0 |
| Potamolinea | 38278 | 65 | 0 |
| Pycnacronema | 728312 | 806 | 55 |
| Schizothrix | 435833 | 747 | 826 |
| Scytonema | 1107826 | 3834 | 55 |
| Tolypothrix | 65012 | 1604 | 0 |
| Unidentified | 37467 | 73 | 0 |
| Wilmottia | 9010 | 0 | 0 |
| Total | 7,939,724 | 16,197 | 2,506 |
